# Supplementary material for: Cell cycle entry triggers a switch between two modes of Cdc42 activation during yeast polarization
Source: eLife. 2017 Jul 6;6:e26722. doi: 10.7554/eLife.26722 (PMC5536948; doi:10.7554/eLife.26722)
Supplement: Supplementary file 1. — DOI: http://dx.doi.org/10.7554/eLife.26722.045 [file elife-26722-supp1.docx]

**Table S1: Strain List**

| **Strain** | **Genotype** |
| --- | --- |
| WYK8301 | TRP1∆::Gal4-rMR1/+; pGal-pGal-Mid2-GFP-LOVpep::LEU2/+; Bem1-tdTomato::HIS3MX/+; pGal-Cdc24-ePDZb1::URA/pADH1-Gal4-VP16-ER::URA; rsr1∆::TRP1/rsr1∆::KanMX |
| WYK8308 | TRP1∆::Gal4-rMR1/+; Gic2(1-208)-tdTomato::HIS3MX/+; pGal-Mid2-GFP-LOVpep::LEU2/+; pGal-Bem1-ePDZb1::URA/pADH1-Gal4-VP16-ER::URA; rsr1∆::TRP1/rsr1∆::KanMX |
| WYK8318 | TRP1∆::Gal4-rMR1/+; Bem1-tdTomato::HIS3MX/+; pGal-Mid2-GFP-LOVpep::LEU2/+; pGal-Bem1-ePDZb1::URA/pADH1-Gal4-VP16-ER::URA; rsr1∆::TRP1/ rsr1∆::KanMX |
| WYK8410 | rdi1∆::KanMX/rdi1∆::HygR; rsr1∆::TRP1/ rsr1∆::TRP1; Bem1-tdTomato::HIS3MX/+; pGal-Mid2-GFP-LOVpep::LEU2/+; pGal-Cdc24-ePDZb1::URA/pADH1-Gal4-VP16-ER::URA; |
| WYK8434 | TRP1∆::Gal4-rMR1/+; Gic2(1-208)-tdTomato::HIS3MX/+; pGal-Mid2-GFP-LOVpep::LEU2/+; pGal-Bem1(R369A)-ePDZb1::URA/ pADH1-Gal4-VP16-ER::URA; rsr1∆::TRP1/ rsr1∆::KanMX |
| WYK8435 | TRP1∆::Gal4-rMR1/+; Gic2(1-208)-tdTomato::HIS3MX/+; pGal-Mid2-GFP-LOVpep::LEU2/+; pGal-Bem1(P355A)-ePDZb1::URA/pADH1-Gal4-VP16-ER::URA; rsr1∆::TRP1/ rsr1∆::KanMX |
| WYK8436 | TRP1∆::Gal4-rMR1/+; Gic2(1-208)-tdTomato::HIS3MX/+; pGal-Mid2-GFP-LOVpep::LEU2/+; pGal-Bem1(K482A)-ePDZb1::URA/pADH1-Gal4-VP16-ER::URA; rsr1∆::TRP1/rsr1∆::KanMX |
| WYK8437 | TRP1∆::Gal4-rMR1/+; Gic2(1-208)-tdTomato::HIS3MX/+; pGal-Mid2-GFP-LOVpep::LEU2/+; pGal-Cdc24∆PB1-ePDZb1::URA/pADH1-Gal4-VP16-ER::URA; rsr1∆::TRP1/rsr1∆::KanMX |
| WYK8439 | TRP1∆::Gal4-rMR1/+; Gic2(1-208)-tdTomato::HIS3MX/+; pGal-Mid2-GFP-LOVpep::LEU2/+; pGal-Cdc24(Q412A R416E L419A)-ePDZb1::URA/pADH1-Gal4-VP16-ER::URA; rsr1∆::TRP1/rsr1∆::KanMX |
| WYK8440 | TRP1∆::Gal4-rMR1/+; Gic2(1-208)-tdTomato::HIS3MX/+; pGal-Mid2-GFP-LOVpep::LEU2/+; pGal-Cdc24-ePDZb1::URA/pADH1-Gal4-VP16-ER::URA; rsr1∆::TRP1/rsr1∆::KanMX |
| WYK8441 | TRP1∆::Gal4-rMR1/+; Bem1-tdTomato::HIS3MX/+; cdc28as::HygR/cdc28as::HygR; pGal-Mid2-GFP-LOVpep::LEU2/+; pGal-Cdc24-ePDZb1::URA/pADH1-Gal4-VP16-ER::URA; rsr1∆::TRP1/rsr1∆::KanMX |
| WYK8442 | TRP1∆::Gal4-rMR1/+; Gic2(1-208)-tdTomato::HIS3MX/+; pGal-Mid2-GFP-LOVpep::LEU2/+; cdc28as:HygR/cdc28as::HygR; pGal-Cdc24-ePDZb1::URA/pADH1-Gal4-VP16-ER::URA; rsr1∆::TRP1/rsr1∆::KanMX |
| WYK8476 | TRP1∆::Gal4-rMR1/+; pGal-Mid2-GFP-LOVpep::LEU2/+; rsr1∆::TRP1/rsr1∆::KanMX; pTEF-ePDZb1-mCherry::URA/pADH1-Gal4-VP16-ER::URA |
| WYK8500 | TRP1∆::Gal4-rMR1/+; Gic2(1-208)-tdTomato::HIS3MX/+; pGal-Mid2-GFP-LOVpep::LEU2/+; pGal-Cdc24-ePDZb1::URA/pADH1-Gal4-VP16-ER::URA; Whi5-tdTomato:HIS3MX/+; rsr1∆::TRP1/rsr1∆::KanMX |
| WYK8502 | TRP1∆::Gal4-rMR1/+; pGal-Mid2-GFP-LOVpep::LEU2/+; Bem1-tdTomato::HIS3MX/+; pGal-Cdc24-ePDZb1::URA/pADH1-Gal4-VP16-ER::URA; Whi5-tdTomato:HIS3MX/+; rsr1∆::TRP1/rsr1∆::KanMX |
| WYK8504 | TRP1∆::Gal4-rMR1/+; pGal-Mid2-GFP-LOVpep::LEU2/+; rsr1∆::TRP1/rsr1∆::KanMX; pGal-Cdc24(Q412A R416E L419A)-ePDZb1::URA/pADH1-Gal4-VP16-ER::URA; pCdc24-Cdc24-tdTomato::HIS3 (pKW101) |
| WYK8505 | TRP1∆::Gal4-rMR1/+; pGal-Mid2-GFP-LOVpep::LEU2/+; Bem1-tdTomato::HIS3MX/+; pGal-Bem1(K482A)-ePDZb1::URA3/pADH1-Gal4-VP16-ER::URA3; rsr1∆::TRP1/rsr1∆::KanMX |
| WYK8550 | Bem1-GFP::HIS3MX/+; pADH1-Gal4-VP16-ER::URA/+; Gic2(1-208)-tdTomato::HIS3MX/+; rsr1∆::TRP1/rsr1∆::KanMX |
| WYK8551 | TRP1∆::Gal4-rMR1/+; Bem1-tdTomato::HIS3MX/+; pADH1-Gal4-VP16-ER::URA/+; pCdc24-Cdc24-GFP::LEU2 (pKW102); rsr1Δ::TRP/rsr1∆::KanMX |
| WYK8552 | pADH1-Gal4-VP16-ER::URA/+; Gic2(1-208)-tdTomato::HIS3MX/+; rsr1∆::TRP/ rsr1∆::KanMX; pCdc24-Cdc24-GFP::LEU2 (pKW102) |
| WYK8553 | TRP1∆::Gal4-rMR1/+; rsr1∆::TRP1/rsr1∆::KanMX; pCdc24-Cdc24-tdTomato::HIS3 (pKW101); ; pCdc24-Cdc24-GFP::LEU2 (pKW102) |
| WYK8554 | TRP1∆::Gal4-rMR1/+; rsr1∆::TRP1/rsr1∆::TRP1; pGal-Mid2-GFP-LOVpep::LEU2/+; pGal-Bem1(K482A)-ePDZb1::URA/+; Bem1-tdTomato::HIS3MX/Bem1-GFP::HIS3MX |
| WYK8575 | TRP1∆::Gal4-rMR1/+; pGal-Mid2-GFP-LOVpep::LEU2/+; pGal-Cdc24-ePDZb1::URA/pADH1-Gal4-VP16-ER::URA; rsr1∆::TRP1/rsr1∆::KanMX; pCdc24-Cdc24-tdTomato::HIS3 (pKW101) |
| WYK8576 | TRP1∆::Gal4-rMR1/+; pGal-Mid2-GFP-LOVpep::LEU2/+; pGal-Bem1-ePDZb1::URA/pADH1-Gal4-VP16-ER::URA; rsr1∆::TRP1/rsr1∆::KanMX; pCdc24-Cdc24-tdTomato::HIS3 (pKW101) |
| WYK8598 | TRP1∆::Gal4-rMR1/+; pGal-Mid2-GFP-LOVpep::LEU2/+; Gic2(1-208)-tdTomato::HIS3MX/+; pGal-Cdc24-ePDZb1::URA/pADH1-Gal4-VP16-ER::URA; rsr1∆::KanMX/+ |
| WYK8599 | TRP1∆::Gal4-rMR1/+; pGal-Mid2-GFP-LOVpep::LEU2/+; Gic2(1-208)-tdTomato::HIS3MX/+; pGal-Bem1-ePDZb1::URA/pADH1-Gal4-VP16-ER::URA; rsr1∆::KanMX/+ |
| All strains are *MATa/MATα* diploids constructed in the W303 background. They have the following additional markers: *leu2-3,112 trp1-1 can1-100 ura3-1 ade2-1 his3-11,15* | |

**Table S2: Plasmid List**

| **Plasmid Name** | **Type** | **Contents** | **Marker** | **Source** |
| --- | --- | --- | --- | --- |
| DLB3299 | pFA6a | tdTomato | HIS3MX | Longtine *et al* 1998 |
| pDS221 | YIplac | pTEF-ePDZb1-mCherry | URA3 | Strickland *et al* 2012 |
| pDS300 | YIplac | pGal-Cdc24-ePDZb1 | URA3 | Strickland *et al* 2012 |
| pDS311 | YIplac | pGal-Cdc24∆PB1-ePDZb1 | URA3 | This Study |
| pDS343 | YIplac | pGal-Mid2-GFP-LOVpep250 | LEU2 | This Study |
| pDS357 | YIplac | pGal-Bem1-ePDZb1 | URA3 | This Study |
| pDS377 | YIplac | pGal-Bem1(R369A)-ePDZb1 | URA3 | This Study |
| pDS381 | YIplac | pGal-Bem1(P355A)-ePDZb1 | URA3 | This Study |
| pDS382 | YIplac | pGal-Bem1(K482A)-ePDZb1 | URA3 | This Study |
| pKW29 | YIplac | pGal-Cdc24(Q412A R416E L419A)-ePDZb1 | URA3 | This Study |
| pKW50 | pFA6a | cdc28-as1 | HygR | This Study |
| pKW101 | pRS | pCdc24-Cdc24-tdTomato | HIS3 | This Study |
| pKW102 | pRS | pCdc24-Cdc24-GFP | LEU2 | This Study |
| pELW886 | pRS | cdc28-as1 | URA3 | Bishop *et al* 2000 |
| pELW909 | YIplac | pADH1-Gal4-VP16-ER | URA3 | Louvion *et al* 1993 |
